# Perceptions of COVID-19 and the Use of Health Information Technology Among People Who Are Uninsured: Multimethod Survey Study

**DOI:** 10.2196/45349

**Published:** 2023-07-28

**Authors:** Khushi S Patel, Cynthia F Corbett, Elizabeth M Combs, Sara B Donevant, Margaret J Selph, Lynette M Gibson, Robin M Dawson, Amit P Sheth, Ronda G Hughes

**Affiliations:** 1 Arnold School of Public Health University of South Carolina Columbia, SC United States; 2 South Carolina Honors College University of South Carolina Columbia, SC United States; 3 Advancing Chronic Care Outcomes through Research & INnovation Center College of Nursing University of South Carolina Columbia, SC United States; 4 College of Nursing University of South Carolina Columbia, SC United States; 5 The Free Medical Clinic Columbia, SC United States; 6 Mary Black College of Nursing University of South Carolina - Upstate Spartanburg, SC United States; 7 Artificial Intelligence Institute University of South Carolina Columbia, SC United States; 8 American Hospital Association Chicago, IL United States

**Keywords:** COVID-19, medically uninsured, medical informatics, telemedicine, mobile apps, health literacy, mobile phone

## Abstract

**Background:**

As of May 2023, the novel SARS-CoV-2 has claimed nearly 7 million lives globally and >1.1 million lives in the United States. Low-income populations are often disproportionately affected by risk factors such as lifestyle, employment, and limited health literacy. These populations may lack the knowledge of appropriate infection precautions or have reduced access to care during illness, particularly in countries without universal health care.

**Objective:**

We aimed to explore the perceptions and experiences of COVID-19, including symptoms and risk factors among uninsured individuals seeking care at a free medical clinic, and to obtain respondents’ perceptions of and suggestions for adapting a mobile health (mHealth) app to an uninsured population known to have low health literacy.

**Methods:**

We conducted a prospective multimethod survey study with a convenience sample of uninsured adults seeking care at 3 free clinics in the United States. Respondents were questioned about their risk for and awareness of COVID-19 symptoms, COVID-19 testing, current technology use, and the use of technology to facilitate their health regarding COVID-19. Data were analyzed using descriptive statistics (eg, frequencies and mean differences). In addition, a small subset of respondents from one of the clinics (n=10) participated in interviews to provide feedback about the design of a COVID-19 web-based smartphone (mHealth) app.

**Results:**

The survey respondents (N=240) were 53.8% (n=129) female, were primarily White (n=113, 47.1%), and had a mean age of 50.0 (SD 11.67; range 19-72) years. Most respondents (162/222, 73%) did not think that they were at risk for COVID-19. Although respondents reported only moderate confidence in their knowledge of the short- and long-term symptoms of COVID-19, their knowledge of the symptoms aligned well with reports published by the Centers for Disease Control and Prevention of the most common acute (590/610, 96.7%) and long-term (217/271, 80.1%) symptoms. Most respondents (159/224, 71%) reported an interest in using the mHealth app to gain additional information regarding COVID-19 and available community resources. Respondents who were interviewed provided suggestions to improve the mHealth app but had overall positive perceptions about the potential usefulness and usability of the app.

**Conclusions:**

It was encouraging that the knowledge of COVID-19 symptoms aligned well with the reports published by the Centers for Disease Control and Prevention and that respondents were enthusiastic about using an mHealth app to monitor symptoms. However, it was concerning that most respondents did not think they were at a risk of contracting COVID-19.

## Introduction

### Background

The novel SARS-CoV-2 causing COVID-19 first emerged in the Wuhan district of China in late 2019 [1]. It progressed into an international pandemic, altering lives around the world [1]. Despite the World Health Organization’s (WHO) declaration of the end of the pandemic on May 5, 2023, nearly 2000 people worldwide continue to perish because of COVID-19 each week [2]. As of May 2023, COVID-19 has claimed nearly 7 million lives globally and >1.1 million lives in the United States alone [2,3]. Symptoms of viral infection include fever, chills, cough, shortness of breath, fatigue, muscle aches, and loss of taste or smell [4]. More severe COVID-19 symptoms and an increased risk of hospitalization and death are common in populations with preexisting conditions, older adults or those who are immunocompromised [1]. In addition, millions are still living through the long-term effects of COVID-19, known as post–COVID-19 condition (PCC; colloquially known as long COVID-19), which is characterized by its own symptomology [5]. The scope of the COVID-19 pandemic highlights population health inadequacies, health care system gaps, health inequities, and challenges in effectively preventing the spread of a virus in which 80% of individuals do not exhibit any symptoms [6,7]. Susceptible, low-income, and underserved populations require resources to help them combat communicable diseases, such as COVID-19, more so than others [1,8,9].

### Low-Income Populations

#### Overview

Studies exploring the relationship between the social and structural determinants of health and the COVID-19 pandemic have shown a socioeconomic gradient in the outbreak of the pandemic wherein groups with lower income levels have a higher incidence of COVID-19 [10]. These trends are not limited to certain areas but are applicable to the broader systems of different nations and different economic systems. Hospital reports from areas such as the Bronx in New York City mirror data from the Biobank in the United Kingdom and incidences across different financial districts in Barcelona, which indicate that highly populated areas consistently experienced more COVID-19 outbreaks [10]. Preventing and mitigating the spread of communicable diseases among low-income populations is a major challenge across the world [11].

#### Lifestyle and Employment

Many COVID-19 prevention guidelines reflect social distancing and activities to reduce individual vulnerability to the virus. However, among low-income populations and disadvantaged individuals, managing health and preventing the spread of an airborne disease can be difficult owing to lifestyle situations. People with low socioeconomic status are often employed in positions that do not offer benefits, such as paid sick time or health insurance [12]. Fearing job loss, these individuals are more likely to work when ill, increasing the risk of transmission [8,13]. They may also be employed in positions classifying them as “essential workers,” such as home health aides and grocery store clerks [14]. By definition, the people employed in these positions are more likely to work through the pandemic, are less able to practice physical distancing, and may have limited access to personal protective equipment [14]. Low-income populations also report a higher percentage of household crowding, defined as households with more than 1 person per room (excluding hallways and bathrooms and including dining rooms, kitchens, and living rooms) [14]. More densely populated living situations can make self-isolation impossible and lead to a higher risk of transmission.

#### Health Literacy

Low-income individuals are more likely to have limited health literacy (LHL) [15]. The WHO defines health literacy as the “ability of individuals to gain access to, understand, and use information in ways which promote and maintain good health for themselves, their families, and their communities” [16]. Health literacy has been primarily measured in high-income countries, all of which have notably prevalent levels of LHL. Approximately 36% of adults in the United States, 40% in England, and 60% in Australia have been reported to have LHL [17]. However, LHL is thought to be even more ubiquitous in lower socioeconomic populations, including lower-income countries, than in higher socioeconomic populations, serving only to further the gap between the 2 groups [17-19]. Individuals with LHL are generally part of racial and ethnic minority groups, are less healthy, are older, and have cognitive impairments [15]. For example, in the United States, LHL is most common in the following groups: American Indian and Alaska Native (48%), Black (58%), and Hispanic (66%) individuals and those aged 65 years (59%) [19]. LHL is also commonly present in populations with low education levels, those living below the poverty line, and those who did not speak English before starting school [19]. The lack of health literacy not only leads to poorer health outcomes but also affects health care access, quality, and patient safety [19]. Even when factors such as health insurance coverage, employment, race, ethnicity, and cognitive function are controlled, individuals with LHL are less likely to seek care in a timely manner, if at all [15].

#### Digital Health Literacy

Despite health literacy challenges, technology-mediated care grew exponentially during the COVID-19 pandemic [20]. Web-based information platforms are easy to access and provide generous amounts of information, so the public has increasingly begun to use these avenues to gather health information [18]. However, many people lack the health literacy skills required to correctly evaluate and understand the information they procure [18]. Health literacy aside, it is also important to remember that the veracity of information found on the internet remains in question. With a higher incidence of LHL among low-income populations, the web-based information they find and trust may not be accurate, contributing to lower adherence to appropriate infection precautions [12] or knowing when and how to seek care if they are ill [15,21].

Therefore, a mobile health (mHealth) app to monitor and address concerns regarding COVID-19 could be beneficial in mitigating the spread by increasing accurate understanding of the virus [18]. In conjunction with data establishing that racial and ethnic minority groups and groups with lower income levels have a higher incidence of COVID-19, these facts validate the need for low-cost community-based intervention [10,14]. The increased availability of mobile phones prompts the likelihood of mHealth interventions being accessible to underserved populations [22] and promotes itself as a viable intervention in the pathway to bridge gaps in health knowledge, tailor medical and health decisions to patient preferences, increase adherence to treatment recommendations, and improve health outcomes [23-25]. Prior research on using mHealth apps for COVID-19–related public health issues confirms their usefulness in increasing health care confidence and management [26]. These apps can also support health care providers by facilitating communication with patients and improving access to health care services by providing decision-making feedback to patients [27,28].

#### Access to Health Care

Globally, low-income populations have less access to health care [29,30]. In countries without universal health care, uninsured people have been disproportionally affected by COVID-19 and underrepresented in investigations surrounding the pandemic [14,31]. This population is less likely to seek care when ill and is at a higher risk for infection because of risk factors such as limited access to health care and LHL [14,32]. In the United States, some states have restrictive income eligibility requirements (eg, South Carolina) that prevent many low-income individuals from qualifying for state-sponsored insurance coverage (ie, Medicaid) [33]. People who are uninsured already have limited access to health care providers and fewer resources; being uninsured and having low income in South Carolina, a predominately rural state, is more challenging than being in most other states of the United States [34]. South Carolina has an uninsured population rate of 12.7%, which is higher than the national United States rate of 10.4% [35]. Compared with urban populations, rural residents have limited access to quality health care, higher rates of chronic conditions (associated with adverse effects during COVID-19 infection), and often forgo necessary medical treatments [36]. Historically, rural populations also tend to have higher mortality rates during outbreaks of infectious diseases [36].

Uninsured adults who live in South Carolina and other states in the United States with Medicaid-restrictive income eligibility requirements often work in low-income jobs that do not offer insurance. Knowing that their health care bills are typically higher because of full cost calculations without insurance-negotiated discounts, 1 in 5 uninsured people typically forgo needed care [37]. Consequently, owing to the stigma of being uninsured and high health care costs, these adults experience worse health outcomes, higher rates of mortality, and premature death [8,32].

Uninsured individuals are also less likely to be tested for COVID-19, despite having higher incidence of positive results [38]. Although demand for care remains high for the uninsured population, free clinics have not necessarily tested or treated those infected with COVID-19 [39]. Without a usual source of care, people who are uninsured may not know where to go to get tested if they think they have been exposed to COVID-19, thus forgoing testing or care out of fear of having to pay out-of-pocket or face stigma from providers in emergency departments [40]. COVID-19–related changes in federal funding often failed to help the uninsured gain coverage [41], especially because it was not necessarily common knowledge that the Coronavirus Aid, Relief, and Economic Security Act covered hospital costs for COVID-19–related care [42]. Thus, existing health care system inequities (ie, a lack of Medicaid coverage) continue, even among those who are newly uninsured due to pandemic-related job layoffs and furloughs.

Basic mHealth technologies (ie, SMS text messages, cell phone calls, and educational materials) have been successfully implemented among diverse low-income populations, including pregnant women, Spanish-speaking migrant workers and other Latino communities, and homeless youth [43-48]. However, there is a paucity of research in using mHealth apps to reduce disparities and improve health outcomes among people who are uninsured. Contrary to preconceived notions, >75% of people who are uninsured have access to smartphones [23,49,50]; however, there is a lack of mHealth apps that are useful to vulnerable populations, and the fees associated with using mHealth apps are often cost prohibitive [51]. Available apps that monitor COVID-19 related symptoms have not combined education, symptom monitoring, and advice before and after COVID-19 with information about available health care resources [52].

Thus, in countries without universal health care, such as the United States, little is known about those who are uninsured and their willingness to use mHealth to self-manage COVID-19 symptoms, treatments, and safety concerns. This is especially true for people with PCC, which researchers and public health officials are still trying to understand due to its puzzling nature, unclear development, and the large variety of symptoms accompanying it [5]. As COVID-19 infections continue and millions are affected by PCC, there is also dearth of knowledge regarding the effects among the uninsured population, who have significant access barriers to testing, health care services, and up-to-date information and how it may impact their health. As a strategy to address these gaps, we sought to (1) explore the perceptions and experiences of COVID-19, including symptoms and risk factors among uninsured individuals waiting to seek care at a free medical clinic, and (2) obtain uninsured respondents’ perceptions of and suggestions for adapting an mHealth app to an uninsured population known to have LHL.

## Methods

### Ethical Considerations and Informed Consent

The study was reviewed in accordance with the guidelines of the Declaration of Helsinki and received an exemption from human subjects research regulations by the Institutional Review Board of the University of South Carolina (Pro00102203, August 7, 2020, initial mHealth app development; Pro00105756 uninsured population survey and interviews, October 6, 2021). Informed consent was obtained from all the respondents involved in the study.

### Participation

Patients were invited by clinic staff or volunteers to complete a COVID-19 survey as they waited for their appointments. Interested patients were given a gallon-sized Ziploc bag containing a pen and a bottle of hand sanitizer. Patients were allowed to keep these items as compensation regardless of whether they completed the survey. Potential study respondents reviewed a plain-language statement before proceeding with the survey questions, which detailed the voluntary and anonymous design of the study.

### Setting and Respondents

Following institutional review board–exempt determination and approval by collaborating clinic leaders, respondents were recruited from 3 free medical clinics in the midlands and upstate regions of South Carolina in the United States. For this study, the uninsured were defined as adults (aged ≥18 years) without employer-based insurance and who did not qualify for Medicaid or Medicare. Additional inclusion criteria were the ability to read and write in English. Respondents were recruited at 2 free medical clinics between December 2020 and May 2021 and a third clinic between November 2021 and March 2022, as they waited for their visit with health care professionals. A convenience sample (N=240) completed the anonymous survey. In addition, 10 uninsured adults from the midlands-area clinic were recruited to provide their perceptions of a COVID-19 symptom-monitoring mHealth app.

### Materials

Researchers developed a 30-question survey to measure the respondents’ demographic characteristics, COVID-19 and PCC symptom knowledge, risk perceptions, reported precautions, information sources, and access to technology. Respondents were also questioned about their interest in using a COVID-19 symptom-monitoring mHealth app (Multimedia Appendix 1). The survey distributed to the third clinic included an additional 2 questions pertaining to the perceptions of the COVID-19 vaccine and vaccination status. These questions were added to the final survey in accordance with vaccine availability to the public. Consistent with national behavioral surveys (eg, National Health and Nutrition Examination Survey and National Survey on Drug Use and Health), the survey was designed to obtain discrete information rather than information that represented specific constructs. Therefore, the survey was not psychometrically evaluated.

**Figure 1:**
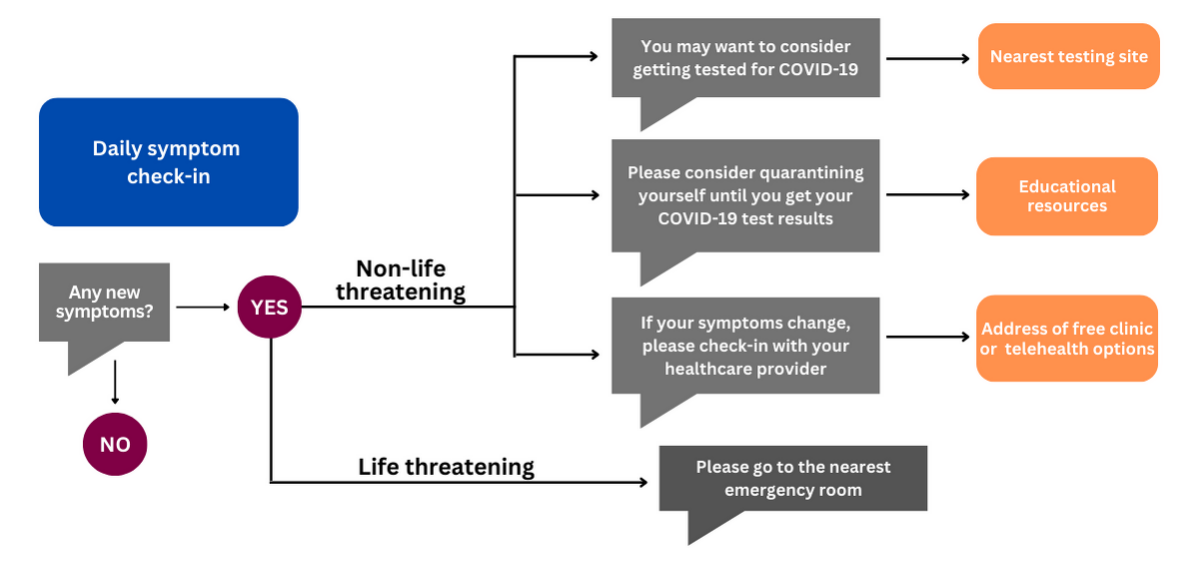
An example of the app engagement flow.

The initial COVID-19 web-based mHealth app was collaboratively developed by health and computer science researchers for use by the university population. The faculty, staff, and student end users provided input and evaluation throughout the development process. In addition to the app, the system includes a dashboard, integrated information, educational materials, and information about health care resources. When users first access the app, they are asked several demographic- and health-related questions. During subsequent access, users are asked whether they have any new or unusual physical symptoms, mental health symptoms, or concerns about their personal safety. If a symptom or concern is indicated, a chatbot guides users through a series of questions using an artificial intelligence algorithm to quickly identify at-risk individuals. On the basis of their responses, users are prompted for next steps. The next steps could include a recommendation to isolate at home, seek health care, or obtain other services when safety is a concern. For situations that seem urgent, users are advised to contact emergency services, such as 9-1-1 or a hotline (eg, mental health and domestic violence), and a link to the recommended service is provided. When users indicate that they do not have any new or unusual symptoms or safety concerns, the chatbot responds with rotating messages, such as “I’m glad you are feeling well,” “It’s a great day to enjoy life,” and “Thank you for washing your hands to prevent the spread of COVID-19.” Data from the mHealth app and chatbot communications were compiled and tracked on a dashboard for efficient, population-based remote monitoring using a secure server. The COVID-19 app’s engagement flow is shown in Figure 1, which details how users would interact with the app’s interface. In this study, the goal of aim 2 was to tailor the mHealth app to a low-income, uninsured population who were likely to have LHL in comparison with the university community end users who contributed to developing the initial app.

### Procedures

Data for aim 1 were obtained via anonymous surveys completed by the patients at the clinic. Volunteer respondents deposited their surveys at a specified collection area in each clinic. Completed surveys were obtained from each clinic by a member of the research team.

To obtain data for aim 2, researchers conducted interviews with patients at the Midland’s clinic on 3 days between January and April 2021. The clinic staff asked patients who were waiting for their clinic visit if they were interested in viewing an mHealth app about COVID-19 and providing feedback on its potential usefulness, usability, and acceptability. Patients who indicated willingness to provide feedback were escorted to the private room by a clinic volunteer or staff member who introduced the patient to the researchers. The research team provided information about the study purpose, potential risks, and benefits and received verbal informed consent from each volunteer respondent (n=10). To elicit think-aloud feedback from the respondents, the COVID-19 mHealth app was displayed on a large screen. The rationale for displaying it on a large screen versus having respondents view it on a mobile phone was 2-fold. First, the community transmission of COVID-19 was high during this part of the research, so physical distancing was required, preventing the ability of the respondent and the researchers to simultaneously view a mobile phone screen. Second, the large screen allowed the researchers to systematically ask the respondents protocol-guided questions on each app screen before moving to the next app screen. The protocol included eliciting each respondent’s think-aloud feedback regarding their understanding of the app content (literacy) and their perceptions of the flow and the cultural appropriateness of the app content. Respondents received a US $10 gift card, a hand sanitizer, and a packet of sanitizing wipes as incentives.

### Analysis

Survey data (aim 1) were analyzed using frequencies, means, and correlations in the SPSS (version 28.0; IBM Corp) statistical database. Respondent-reported COVID-19 short-term and long-term symptoms were compared with short-term and long-term symptom lists published by the Centers for Disease Control and Prevention (CDC) to determine the accuracy of respondents’ symptom knowledge. To achieve aim 2, respondents’ qualitative responses were recorded using field notes. Detailed notes about the respondents’ interview responses were then qualitatively analyzed for response trends using low-inference content analysis [53].

## Results

### Survey (Aim 1)

#### Demographics

The sample’s (N=240) mean age was 50.0 (SD 11.67; range 19-72) years. More than half (129/239, 54%) of the patients were female. The majority were Black (103/240, 42.9%) or White (113/240, 47.1%), with the remaining identifying as American Indian, Indigenous, or Alaska Native (13/240, 5.4%); Asian (2/240, 0.8%); Hispanic, Latino or Latina, or of Spanish origin (19/240, 7.9%); and “other” (2/240, 0.8%). There were no statistically significant differences in the results across racial, ethnic, or gender groups. Of the total sample, 49.1% (115/234) had been tested for COVID-19 with 13.7% (16/117) testing positive, 82.1% (96/117) testing negative, and 4.3% (5/117) reporting an “unknown” result. The reasons for being tested included having symptoms (35/103, 34%), doing so as a precaution (25/103, 24.3%), being exposed to someone who was COVID-19 positive (20/103, 19.4%), being hospitalized for unrelated treatment (5/103, 4.9%), and returning to work or school (10/103, 9.7%).

#### COVID-19 Symptom Knowledge

When asked to rank how well they understood the short-term and long-term COVID-19 symptoms, the answers were generally dispersed across all choices. In terms of short-term COVID-19 symptoms, 9% (20/221) selected “not at all,” 10.9% (24/221) selected “very little,” 19.9% (44/221) selected “neutral,” 26.2% (58/221) selected “somewhat,” and 33.9% (75/221) selected “completely.” In terms of long-term COVID-19 symptoms, 15.8% (35/222) selected “not at all,” 15.8% (35/222) selected “very little,” 23% (51/222) selected “neutral,” 23% (51/222) selected “somewhat,” and 22.5% (50/222) selected “completely.” The most common responses when questioned regarding short-term COVID-19 symptoms were fever, cough, and shortness of breath. The most common long-term symptoms reported were respiratory issues and death (Table 1). When these symptoms were compared with information from the CDC website, 96.7% (590/610) of respondent-reported short-term symptoms and 80.1% (217/271) of respondent-reported long-term symptoms aligned with information published by the CDC.

**Table 1:** Reported long- and short-term symptoms (N=240).

Symptom			Sample, n (%)
**Short-term symptoms**			
	Fever	122 (50.8)	
	Cough	81 (33.8)	
	Shortness of breath	68 (28.3)	
	Loss of taste	67 (27.9)	
	Loss of smell	52 (21.7)	
	Body or chest ache	35 (14.6)	
	Headache	35 (14.6)	
	Nose or sinus issues	29 (12.1)	
	Fatigue	22 (9.2)	
	Flu-like symptoms	21 (8.8)	
	Chills	21 (8.8)	
	Sore throat	15 (6.3)	
	Vomiting or nausea	12 (5)	
	Diarrhea	10 (4.2)	
	Other	20 (8.3)	
**Long-term symptoms**			
	Respiratory issues	45 (18.8)	
	Death	31 (12.9)	
	Unknown	30 (12.5)	
	Loss of taste	24 (10)	
	Cardiovascular issues	23 (9.6)	
	Shortness of breath	20 (8.3)	
	Loss of smell	20 (8.3)	
	Fatigue	13 (5.4)	
	Neural issues	13 (5.4)	
	Fever	10 (4.2)	
	Other	44 (18.3)	

#### COVID-19 Risk Perceptions and Reported Precautions

Most survey respondents (162/222, 73%) stated that they were not at risk of COVID-19, with only 27% (60/222) stating that they were at risk. When asked to elaborate on their answers, the responses generally fell into two categories: (1) factors associated with being at risk for COVID-19 and (2) factors associated with not being at risk for COVID-19. Respondents who thought they were at risk for COVID-19 reasoned so because they were unable to isolate or maintain social distance for various reasons (32/222, 14.4%) or they had preexisting health conditions (18/222, 8.1%). Respondents who said they were not at risk reported the following reasons: taking precautions (such as masking or handwashing) to prevent infection (33/222, 14.9%), practicing isolation (40/222, 18%), young or generally healthy (18/222, 8.1%), being vaccinated (11/198, 5.6% at clinics 1 and 2 when vaccines were not widely available and 19/222, 8.6% at clinic 3 when vaccines were widely available), and being regularly tested for COVID-19 (4/222, 1.8%). Respondents were also asked an open-ended question on the precautions they had taken against the pandemic. The main precautions reported by the respondents included wearing a mask (67/222, 30.2%), self-isolation (51/222, 23%), social distancing (32/222, 14.4%), and regular hand washing (21/222, 9.5%). Notably, 8.1% (18/222) of the respondents reported that they did not take any precautions to prevent COVID-19 (Table 2).

**Table 2:** COVID-19 precautions overall and according to risk perception.

Reported precaution	Total sample (n=222), n (%)^a^	Perception of at risk for COVID-19 (n=60), n (%)^a^	Perception of not at risk for COVID-19 (n=162), n (%)^a^
Mask	67 (30.2)	35 (58.3)	42 (25.9)
Isolation	51 (23)	15 (25)	36 (22.2)
Social distance	32 (14.4)	13 (21.7)	19 (11.7)
Wash hands	21 (9.5)	10 (16.7)	11 (6.8)
Prayer or faith	15 (6.8)	2 (3.3)	13 (8)
Sanitizer	14 (6.3)	6 (10)	8 (4.9)
Awareness of environment or self	8 (3.6)	2 (3.3)	6 (3.7)
Follow precautions or protocol	6 (2.7)	1 (1.7)	5 (3.1)
Talk to people	4 (1.8)	0 (0)	4 (2.5)
None	18 (8.1)	6 (10)	12 (7.4)

^a^The total is not equal to 100% because respondents listed more than 1 precaution.

When respondents’ perceptions of their risk of COVID-19 were compared with their responses about the precautions taken, 21.6% (48/222) reported they were at risk and took precautions, 2.7% (6/222) reported they were at risk but did not take precautions, 48.2% (107/222) said they were not at risk but took precautions, and 9% (20/222) said they were not at risk and did not take precautions. Table 2 shows the percentage of the risk perception groups engaged in each reported precaution.

When asked what strategies they used to cope with pandemic-related stress and fear, the most notable responses were engaging in a hobby (122/281, 43.4%), self-care or meditation (47/281, 16.7%), taking proper infection precautions (52/281, 18.5%), prayer (26/281, 9.3%), and doing research on the pandemic (12/281, 4.3%). Of the 281 respondents, 11 (3.9%) respondents said they did not take any steps to alleviate these feelings.

#### Vaccination

Only respondents in clinic 3 were surveyed on vaccination status (November 2021 to March 2022), after widespread availability of the COVID-19 vaccine. Overall, 60% (41/68) of respondents from clinic 3 reported being vaccinated, with 37% (25/68) saying they were not vaccinated. When unvaccinated respondents were asked why they had not received the vaccine, the majority (19/25, 76%) said it was because they were worried about the side effects (Table 3).

**Table 3:** COVID-19 vaccine hesitancy.

Reasons for not receiving the COVID-19 vaccine	Subsample of unvaccinated respondents at clinic 3 (n=25), n (%)^a^
I’m worried about the side effects	19 (76)
I do not believe it will prevent COVID-19	12 (48)
I’ve already had COVID-19	3 (12)
My provider told me I should not receive the vaccine due to my health conditions	2 (8)
I do not trust the government	2 (8)
Religious reasons	2 (8)
I see no reason to get vaccinated	2 (8)
I disagree with the pressure to be vaccinated	2 (8)
My family schedule	1 (4)
I don’t know where to get vaccinated	1 (4)
A family member passed away after being vaccinated	1 (4)
My work schedule	0 (0)

^a^The total is >100% because respondents checked all that applied.

#### COVID-19 Information Sources

The information sources for COVID-19 varied greatly within the sample with the following responses: 59.6% (143/240) television, 49.2% (118/240) internet searches, 47.9% (115/240) health care provider, 46.7% (112/240) family, 45.8% (110/240) news feed on a phone or computer, 45% (108/240) friends, and 28.8% (69/240) social media. Most of the respondents (194/240, 80.8%) reported using multiple sources of information.

#### Access to Technology and Sentiment Toward Using a COVID-19 App

In terms of accessibility, 82.3% (190/231) of the respondents had access to a smartphone, 53.2% (118/222) had access to a computer, and 87.7% (207/236) had access to the internet via a phone or computer. Of the respondents who had access to a smartphone, 68.7% (90/131) had an Android and 31.3% (41/131) had an iPhone. Few respondents with access to technology used it to track their health, with 17.9% (37/207) using a computer, 24.6% (51/207) using a smartphone, and 26.1% (54/207) using some form of the internet to track health (Table 4). There was an overall positive response for the use of a COVID-19–related mHealth app, with nearly three-quarters (159/224, 71%) of the respondents stating that they would use an mHealth app to learn more about COVID-19 symptoms and to help them know when to get tested.

**Table 4:** Most common smartphone and computer uses among respondents with device access.

Device		Sample, n (%)
**Smartphone (n=207)**		
	Access the internet	155 (74.9)
	Access news	111 (53.6)
	Access social media	127 (61.4)
	Look up health information	110 (53.1)
	Make phone calls	169 (81.6)
	Receive emails	126 (60.9)
	Set reminders for appointments or tasks	106 (51.2)
	Send SMS text messages	153 (73.9)
	Track health	51 (24.6)
**Computer function (n=146)**		
	Access the internet	116 (79.5)
	Access news	64 (43.8)
	Access social media	71 (48.6)
	Look up health information	69 (47.3)
	Set reminders for appointments or tasks	45 (30.8)
	Receive emails	84 (57.5)
	Track health	26 (17.8)

### COVID-19 mHealth Symptom-Monitoring App (Aim 2)

The respondents were primarily female (8/10, 80%); in terms of race and ethnicity, 40% (4/10) were Black, 50% (5/10) were White, and 10% (1/10) preferred not to answer. Respondents’ perspectives on the usability of the COVID-19 app were generally positive:

Looks pretty solid.

Respondent 2

Respondents unanimously perceived the flow of the app from one screen to the next to be intuitive. Most respondents also reported that the app would be useful:

I think it would be real helpful.

Respondent 4

Most respondents also stated that they would try the app to see if it was useful and believed that others would as well:

I think the app would be very helpful and that people would use it if they had symptoms and needed advice.

Respondent 8

I am not a “people person” so I would rather get health advice from the internet than talking to someone in person or even on the phone.

Respondent 6

Conversely, 2 respondents indicated that people may have some reservations about the app. Respondent 2 stated that she thought there should be a “disclaimer” about the mental health questions as “many people wouldn’t go near it*”* (ie, using an app that asked them to report mental health symptoms). Respondent 3 initially provided positive feedback, saying “most people would use it because it is about a topic they might not know about,*”* but then seemed to warn about potential barriers:

There is a narrative out there—that the CDC will track you after you get the vaccine...There is a lot of conspiracy theory out there.

Respondent 3

Specific features of the COVID-19 app that respondents commented on favorably were the frequently asked questions; the app’s “checkback” feature, which involved the app initiating follow-up text questions after a certain period when the user reported a symptom; and that using the app could save people time by giving advice about the need for follow-up care so as to avoid waiting to be seen by health care professionals if an in-person consultation was not necessary. The respondents offered 2 valuable suggestions for improving the health literacy of the app. They advised changing “environmental allergies” to “seasonal allergies (eg, pollen)” and “digestive” to “stomach or intestine.” One respondent proposed additional content for the frequently asked questions, and some respondents suggested including a section in the app for general health monitoring rather than limiting the app to COVID-19specific conditions.

## Discussion

### Principal Findings

Respondents showed accurate knowledge of the short-term and long-term COVID-19 symptoms. When asked to rank their understanding of COVID-19 symptoms on a scale of 1 to 5, the average ranking for short-term symptoms was 3.7 and the average ranking for long-term symptoms was 3.2. However, when self-reported short-term and long-term symptoms were compiled and compared with the information reported on the CDC website, the results showed a very high level of understanding: 97% of reported short-term symptoms and 81% of reported long-term symptoms aligned with information published by the CDC [4]. Respondents may have been unsure of their knowledge because of varied reports and evidence of additional symptoms as the pandemic progressed. However, this finding may also suggest that although people know accurate disease information, they may not be confident in their knowledge, an interpretation that is consistent with the WHO definition of LHL. According to the WHO, health literacy requires that people not only understand information but also have the ability to act on the information to promote and maintain their health [16]. The respondents’ lack of confidence in their own knowledge could lead to delays in seeking health care, as recognition of short-term symptoms is key in identifying COVID-19 infections and taking proper precautions to get tested and self-isolate.

When questioned about their risk of infection, only about one-quarter (60/222, 27%) of the respondents considered themselves to be at risk for COVID-19. Of the participants who reported that they were not at risk, half (104/198, 52.5%) indicated that they were taking some form of precaution to mitigate risk. They reasoned that participating in behaviors such as masking, social distancing, handwashing, and isolation equated to a very low risk of infection, or none at all. Although these behaviors reduce the chances of contracting COVID-19, they are not a definite solution for preventing infection [54]. Of the group who said they were not at risk and were not taking precautions against COVID-19, the most common rationale for their reasoning was that they were young or generally healthy; thus, they would not contract the virus. In essence, although most of the respondents understood behaviors that could reduce their risk for COVID-19, there were misconceptions regarding the spread of COVID-19, particularly among groups who thought themselves not at risk of infections because they were “young” or “generally healthy.” To address this, the mHealth app proposed in this study could additionally be used to fully educate users on their risk of infection.

### Pandemic Information Sources

When examining pandemic information sources, most respondents were not receiving their knowledge from a health care provider. Instead, they used television or internet searches as their primary source of information. Although correlational analysis revealed no strong relationship between information sources and the knowledge of COVID-19, this is concerning from a health care perspective, as research conducted on pandemic information sources has found that people who relied on television as their primary source were less knowledgeable about the virus than groups whose primary sources were government websites [55]. In addition, with most people quarantined in their homes during a digital age, the internet has become a popular source for pandemic information and misinformation [56]. The accuracy of internet search results depends on the source; many web-based resources are unchecked and are likely to spread incorrect details [57]. In a population with LHL, such as those who are uninsured, relying heavily on reports on the internet may be dangerous, especially in the context of a rapidly expanding pandemic. Although not a majority, 28.8% (69/240) of respondents received knowledge from social media, which is known to propagate misinformation owing to the viral nature of the platform and the ability of anyone to post anything [58,59].

Concern about attaining accurate information leads to the question: what is the best source of pandemic-related information? Although the easiest answer would be a health care professional, there are many barriers that hinder both uninsured and insured populations from accessing them. First, during the COVID-19 pandemic, many health care providers and facilities were temporarily closed, limiting access to everyone. Second, when health care professionals began to see patients again, higher patient volumes and the need to implement safe distancing led to longer wait times for all individuals seeking care. Third, rising health care costs are barriers, and the uninsured population is both less likely to have a usual source of care and less likely to visit a provider when ill owing to high out-of-pocket costs [60,61]. Thus, an evidence-based mHealth app could fill this information gap for those who are uninsured.

The mHealth app investigated in this study could be used by people at risk of COVID-19 and by those with PCC. Recent research indicates that approximately 6.2% of people around the world have symptoms for 3 months after having COVID-19 [62]. PCC symptoms include debilitating fatigue, shortness of breath, pain, difficulty sleeping, racing heart rate, exercise intolerance, and gastrointestinal and cognitive problems [5]. Much is still unknown about this condition, and considering initiatives currently in place to better understand it (such as the National Institute of Health Researching COVID to Enhance Recovery initiative), this app could aid in monitoring PCC and further documenting the frequency and severity of PCC symptoms and disease trajectory.

The mHealth app described in this study fills a need for people who are uninsured by providing a low-cost intervention with accurate symptom management information that they can use on demand. Smartphone apps have been shown to be useful for understanding outbreak epidemiology, screening individuals, and conducting contact tracing, making them likely fixtures in future epidemics [63]. The functionality of mHealth apps as intervention tools has been tested across many countries globally, and the results show positive outcomes and high levels of support for these types of apps due to improved disease management and easy health information sharing [64-67]. With most uninsured respondents having access to a smartphone, there is high potential for using the COVID-19 app, especially when considering the rapid increase in health care technology during the pandemic [20]. Few respondents (51/207, 24.6%) used their phones to track health; however, research indicates that this number is rapidly growing. The Pew Research Center recently reported that 62% of the population currently uses mHealth devices to gather health-related information [68]. Notably, because the app is web-based, people could access it via a laptop or computer if they did not have a smartphone. Thus, the information is available to those who access the internet with a smartphone or via a computer, laptop, or tablet.

Traditionally, smartphone health app users are characterized as being younger with higher income and education levels, a description not indicative of the uninsured population [69], which may explain the much lower use of apps to track health (51/207, 24.6%) that were reported in this study. However, in the qualitative interviews, respondents provided positive feedback about the app, and most of them indicated that they would use it if it were available. Future research is needed to obtain knowledge about promoting the use of the app and the use and perceived usefulness of the app.

### Limitations

Some respondents did not complete the survey, leaving pages or questions unanswered. In addition, the sample examined in this study was collected from 2 clinics between December 2020 and May 2021 and from a third clinic between November 2021 and March 2022. These are only snapshots of time and are not a holistic indication of the progression of the pandemic. It is important to note that the responses remained constant during the data collection periods. However, respondents from the third clinic had slightly more knowledge of long-term symptoms as compared with respondents from the other 2 clinics, which is likely a result of developing information about long-term symptoms as the pandemic progressed.

### Conclusions

Uninsured respondents had an overall high knowledge of COVID-19 symptoms, and most reported implementing at least some precautionary behaviors to protect themselves and prevent the spread of infection. However, respondents’ confidence in their knowledge was only moderate, suggesting that they may not have the health literacy skills to effectively use their knowledge. Most respondents reported that they would be interested in using a COVID-19 mHealth app to monitor symptoms and access community resources if it were available to them. Respondents who viewed the app features had positive feedback, with some suggestions to improve the language used in the app to make it more comprehensible to the LHL population. Further research will involve testing the app and the website hosting the app. Overall, this study suggests that a web-based mHealth app that provides information about COVID-19 and community resources could be a helpful intervention to reduce the spread of COVID-19 and mitigate the sequelae of COVID-19, including PCC, by providing accurate information and access to resources among those who are uninsured and possibly other at-risk populations.

## Appendix

Multimedia Appendix 1 The full, revised survey with vaccination questions.

## Abbreviations

CDC: Centers for Disease Control and Prevention
LHL: limited health literacy
mHealth: mobile health
PCC: post–COVID-19 condition
WHO: World Health Organization

## Data Availability

Deidentified data supporting the reported results will be available upon request from the corresponding author (CFC).
