# Supplementary material for: Perceptions of COVID-19 and the Use of Health Information Technology Among People Who Are Uninsured: Multimethod Survey Study
Source: JMIR Form Res. 2023 Jul 28;7:e45349. doi: 10.2196/45349 (PMC10389075; doi:10.2196/45349)
Supplement: Multimedia Appendix 1 [file formative_v7i1e45349_app1.docx]

| **Researchers at the University of South Carolina are asking you to help us understand about your experience with COVID-19. We ask that you complete this survey anonymously. Please know that only the researchers will have access to these surveys. No one at the clinic will be able to see the information you provide us.**  **Once you have completed the survey, please place it in the envelope and then put it in the locked box near the receptionist. If you don’t want to answer a question, just move on to the next question. If you don’t want to complete the survey, please follow the same procedures.**    **We appreciate you, your time and interest in helping us.** |
| --- |

**Age: ________**

**Occupation: _________________**

**What is your gender identity?**

**______ Man 
______ Woman**

**______ Open Option: ____________________**

**Ethnicity (choose all that apply):**

**_______ American Indian, Indigenous, or Alaska Native**

**_______ Asian**

**_______ Black or African American**

**_______ Hispanic, Latino/a, or Spanish origin**

**_______ Middle Eastern or North African**

**_______ Native Hawaiian or Other Pacific Islander**

**_______ White**

**_______ Open Option: ____________________**

**How would you identify your sexual orientation?**

**_____ Straight / Heterosexual**

**_____ Lesbian/ Gay/ Homosexual**

**_____ Bisexual**

**_____ Open Option: ____________**

**Please indicate your highest level of education:**

**_____ Less than a High School Diploma**

**_____ High School Diploma or GED equivalent**

**_____ Associates Degree or Certification (Technical College)**

**_____ Bachelor’s degree**

**_____ Master’s and above**

**When the clinic is closed, where do you go to get healthcare? _______________________**

**Have you been tested for COVID 19? Please check one: ____YES ____NO**

**If you have been tested for COVID 19, please rate the experience: (1very unsatisfied, 2 somewhat unsatisfied, 3 neutral, 4 somewhat satisfied, and 5 very satisfied) Please circle one:**

**1 2 3 4 5**

**If you have been tested for COVID 19, why did you get tested? ____________________________________________________________________________________________________________________________________________________________**

**If you have been tested for COVID 19, what were your results? Please check one: ____POSITIVE ____NEGATIVE ____UNKNOWN**

**If you haven’t been tested for COVID 19 and wanted to get tested, what would you do?:**

**____________________________________________________________________________________________________________________________________________________________**

**If you thought that you had COVID 19, would you get tested? Please check one:**

**_____YES _____NO _____UNSURE**

**Do you consider yourself at risk for COVID 19? Please check one:**

**_____YES _____NO**

**Why or why not? ____________________________________________________________________________________________________________________________________________________________**

**Have you gotten the vaccine for COVID 19? Please check one:**

**_____YES _____NO**

**If you haven’t received the COVID-19 shot, please check all reasons that apply:**

**­­­______My work schedule**

**______My family schedule**

**______I don’t know where to get a vaccine**

**______I worry about side effects**

**______I don’t believe it will help prevent COVID**

**______I already had COVID so I don’t think I need a vaccine**

**______My provider told me I should not be vaccinated to due my health condition(s)**

**Other personal beliefs, please specify: ____________________________________________________________________________________________________________________________________________________________**

**Other reasons, please specify: _____________________________________________________________________________________________________________________________________________________**

**Would you use an app on your phone or computer to help you better understand COVID 19 and when it would be good to get tested? Please check one:**

**_____YES _____NO**

**Do you own a smartphone? Please check one:**

**_____YES _____NO**

**If yes, Android or iPhone? _________________________________**

**How frequently do you use your smartphone? Please check one:**

**_______Daily**

**_______Several times a week, but not daily**

**_______Once/week**

**_______Several times a month**

**Which of the following functions do you complete on your smartphone? Please check as many that apply:**

**______Access the Internet**

**______Access news**

**______Access social media**

**______Look up health information Internet**

**______Make phone calls**

**______Send/receive e-mails**

**______Reminders for appointments, tasks**

**______Text**

**______Track your health**

**Do you have access to a computer? Please check one:**

**_____YES _____NO**

**If Yes:**

**How frequently do you use the computer? Please check one:**

**_____Daily**

**_____Several times/week, but not daily**

**_____Once/week**

**_____Several times/month**

**Which of the following do you do on the computer? Please check as many that apply:**

**_____Access the Internet**

**_____Access the news**

**_____Access social media**

**_____Look up health information on the Internet**

**_____Reminders for appointments, tasks**

**_____Send/receive e-mails**

**_____Track your health**

**With the pandemic, more stress and fear are common. What strategies do you use when you are more stressed, fearful, concerned, or feel hopeless? ____________________________________________________________________________________________________________________________________________________________**

**With the pandemic, some people may have more fear for their safety. What steps do you take if you have safety fears?**

**____________________________________________________________________________________________________________________________________________________________**

**How well do you understand the short-term symptoms of COVID 19? (1 not at all, 2 very little, 3 neutral, 4 somewhat, 5 completely) Please circle one:**

**1 2 3 4 5**

**From what you have read and heard, what are common symptoms of COVID 19?**

**____________________________________________________________________________________________________________________________________________________________**

**How well do you understand the long-term risks and symptoms of COVID 19? (1 not at all, 2 very little, 3 neutral, 4 somewhat, 5 completely) Please circle one:**

**1 2 3 4 5**

**From what you have read and heard, what are the long-term risks and symptoms of COVID 19?**

**____________________________________________________________________________________________________________________________________________________________**

**Where do you get your information about COVID 19? (check all that apply)**

**_____Family**

**_____Friends**

**_____Healthcare provider**

**_____Internet searches**

**_____News feeds on phone or computer (including videos)**

**_____TV**

**_____Social media (Facebook, Twitter)**

**_____Other**
